# Overexpression of HTRA1 Leads to Ultrastructural Changes in the Elastic Layer of Bruch's Membrane via Cleavage of Extracellular Matrix Components

**DOI:** 10.1371/journal.pone.0022959

**Published:** 2011-08-02

**Authors:** Sarah Vierkotten, Philipp S. Muether, Sascha Fauser

**Affiliations:** Center of Ophthalmology, University of Cologne, Cologne, Germany; University of Florida, United States of America

## Abstract

Variants in the chromosomal region 10q26 are strongly associated with an increased risk for age-related macular degeneration (AMD). Two potential AMD genes are located in this region: *ARMS2* and *HTRA1* (high-temperature requirement A1). Previous studies have suggested that polymorphisms in the promotor region of *HTRA1* result in overexpression of HTRA1 protein. This study investigated the role of HTRA1 overexpression in the pathogenesis of AMD. Transgenic *Htra1* mice overexpressing the murine protein in the retinal pigment epithelium (RPE) layer of the retina were generated and characterized by transmission electron microscopy, immunofluorescence staining and Western Blot analysis. The elastic layer of Bruch's membrane (BM) in the *Htra1* transgenic mice was fragmented and less continuous than in wild type (WT) controls. Recombinant HTRA1 lacking the N-terminal domain cleaved various extracellular matrix (ECM) proteins. Subsequent Western Blot analysis revealed an overexpression of fibronectin fragments and a reduction of fibulin 5 and tropoelastin in the RPE/choroid layer in transgenic mice compared to WT. Fibulin 5 is essential for elastogenesis by promoting elastic fiber assembly and maturation. Taken together, our data implicate that HTRA1 overexpression leads to an altered elastogenesis in BM through fibulin 5 cleavage. It highlights the importance of ECM related proteins in the development of AMD and links *HTRA1* to other AMD risk genes such as fibulin 5, fibulin 6, *ARMS2* and *TIMP3*.

## Introduction

Age-related macular degeneration (AMD) is the most common cause of central blindness in the elderly population. Both genetic and environmental factors play important roles in the development of AMD. Smoking and dietary intake have been identified as the major environmental risk factors for developing AMD besides age [1], [2]. To identify genetic risk factors of AMD, genome-wide linkage studies had been conducted by several groups [3], [4], [5]. Two major susceptibility loci were identified: at chromosome 1q31, variants in the complement factor H (*CFH*), and variants at chromosome 10q26. These two loci account for more than 50% of the disease risk [6].

Two potential AMD genes reside in this region: *ARMS2* (age-related maculopathy susceptibility 2) and *HTRA1* (high-temperature requirement factor A1). Ever since, there is considerable controversy on which gene plays a causal role in AMD [7], [8], [9], [10]. Strong linkage disequilibrium across the region probably makes genetic studies unsuitable to solve this question. Recently, Tong et al. (2010) [11] suggested that polymorphisms in both genes were genetic risk factors of AMD. Polymorphisms in the promotor region were reported to increase expression levels of HTRA1 [12], [13], although others could not confirm these findings [10], [14].

HTRA1 is a member of a family of serine proteases characterized by a highly conserved trypsin-like protease domain and a C-terminal PDZ domain. A 22 amino acid signal peptide at the N-terminus marks the HTRA1 protein for secretion. It is involved in degradation of extracellular matrix (ECM) proteins like fibronectin [15] and aggrecan [16]. Elevated HTRA1 levels have been associated with arthritic disease [15], [17], [18]. Therefore, it seems to be an important protein of ECM homeostasis and turnover. Reduced HTRA1 activity did not repress signaling by the TGF-ß family and resulted in familial ischemic cerebral small-vessel disease [19], [20], [21]. The involvement of the ECM in the pathogenesis of AMD is further supported by additional AMD risk genes such as *TIMP3* (tissue inhibitor of metalloproteinases-3), which inhibits MMPs (matrix metalloproteinases) and is involved in degradation of the ECM [22], and *ARMS2*, which interacts with several ECM proteins [23].

To date, there is no physiological evidence of HTRA1 involvement in AMD pathogenesis. The aim of this study was the functional analysis of HTRA1 overexpression in the retinal pigment epithelium (RPE) of the mouse eye. Here, we show for the first time that an elevated expression of HTRA1 in RPE cells leads to a change of Bruch's Membrane (BM) composition *in vivo*.

## Results

### Generation of transgenic mice and evaluation of HTRA1 expression

**Figure 1:**
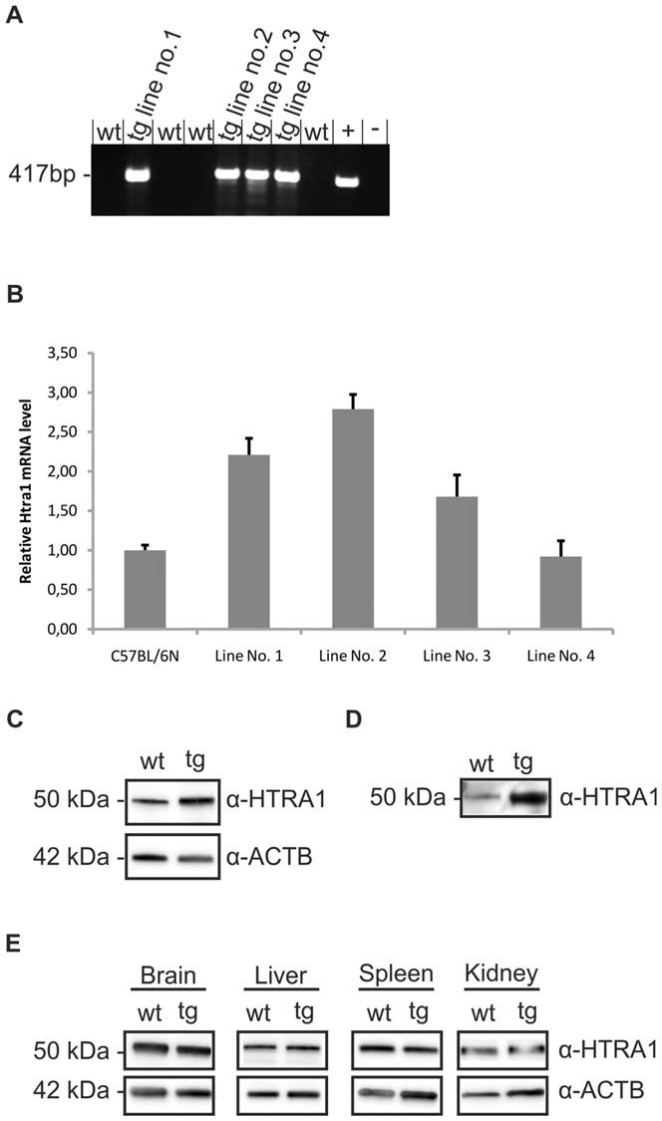
Genotyping and protein analysis of *Htra1* transgenic mice. (A) PCR of genomic DNA from transgenic and WT mice. Transgenic mice show amplification with a visible band at 417 bp (tg) as can be seen in the positive control (+). WT mice (wt) as well as the negative control (−) show no amplification. (B) Quantitative real-time PCR determination of *Htra1* mRNA levels from total RNA isolated from posterior eyes of C57BL/6N and transgenic lines no. 1 to 4 mice. An increase of *Htra1* mRNA in *Htra1* transgenic lines compared to WT was observed. Transgenic line no 2 shows the highest rate with a 2.79 fold increase (3mice/C57BL/6N and 12mice/transgenic line: 3 independent experiments, 3mice/group). Data are expressed as means ±SD. Relative mRNA levels were normalized to *Actb* and expressed as fold change relative to control. (C) Western Blot analysis of RPE/Choroid lysates from 3 month old WT (wt) and transgenic (tg) mice. Transgenic mice show a higher expression of HTRA1 protein compared to WT (1mice/group, 6 independent experiments). Expression of ACTB serves as a loading control. Image is representative. (D) Western Blot analysis of primary RPE cells supernatant. Cells were cultured till confluent and medium was removed 24 hours after exchange of fetal bovine serum-free medium. The experiment clearly demonstrates secretion of HTRA1 from RPE cells (1mice/group, 6 independent experiments). Note that the expression level of HTRA1 is higher in transgenic cell culture supernatants. Image is representative. (E) Western Blot analysis of tissue extracts from 3 month old WT (wt) and transgenic mice (tg). There are no differences in HTRA1 expression found in brain, liver, spleen and kidney of transgenic mice compared of WT (1mice/group, 6 independent experiments). Expression of ACTB serves as a loading control. Image is representative.

Founder mice were generated by microinjection of the transgene into the male pronucleus of one-cell embryos, which were then transplanted into pseudopregnant females that gave birth to founder mice. The expression of the transgenic construct was assessed by PCR on genomic DNA. Transgene-specific primers amplified a transgene cDNA-specific 417-bp sequence in transgenic, but not in the WT mice (Fig. 1A). Sequencing of the transgene showed the correct sequence of the complete Rpe65/Htra1 construct.

To determine the *Htra1* mRNA level of transgenic mice compared to WT, we performed relative quantification by real-time PCR (Fig. 1B). As a control for experimental variability we used beta-actin (*Actb)* as normalizer gene. The fold change in *Htra1* gene expression was calculated with the Pfaffl method and revealed the highest *Htra1* gene expression in transgenic line no. 2 with a 2.79 fold increase compared to WT mice. This is consistent with findings by Yang et al. [13] who demonstrated a 2.7 fold mRNA increase of *HTRA1* in the RPE of patients genotyped for the risk variant. Therefore, our *Htra1* transgenic mice may be regarded as a physiological model of HTRA1 overexpression and may reflect the situation in AMD patients carrying the risk variant. Offspring from line no. 2 mice were then generated by mating a transgenic parent with a C57BL/6N mouse and expanded by at least six back-crosses. Animals were kept heterozygous for the transgene.

Since the mRNA levels do not necessarily correlate with protein levels, we performed Western Blot analysis with RPE/Choroid lysates of transgenic and WT mice to confirm HTRA1 overexpression on protein level. Here, we found moderate expression of HTRA1 protein in C57BL/6N mice, while the *Htra1* transgenic mice showed an increase in expression (Fig. 1C). When we conducted densitometric analysis of our Western Blot experiments, we detected a 2,68-fold overexpression of HTRA1 protein in the transgenic mice compared to WT mice (Fig. S1). Thus, mRNA and protein levels of HTRA1 did correlate in our transgenic mice and both demonstrated a physiological overexpression as seen in AMD patients.

To monitor the secretion of HTRA1, we cultured primary RPE cells from WT and *Htra1* transgenic mice and performed Western Blot analysis of cell culture supernatants (Fig. 1D). Here, we detected HTRA1 in the supernatant of WT as well as transgenic mice. This finding clearly demonstrates the secretion of HTRA1 from primary cultured RPE cells into the medium. Note that the secretion of HTRA1 was increased in *Htra1* transgenic mice.

Furthermore, we evaluated HTRA1 expression in other tissues to demonstrate that the overexpression was restricted to the RPE due to the Rpe65 Promoter, and that HTRA1 overexpression in the RPE was not due to any strain variances. Figure 1E illustrates that no overexpression of HTRA1 protein could be found in brain, liver, spleen or kidney of transgenic mice when compared to WT mice.

### Morphological evaluation of *Htra1* transgenic mice

**Figure 2:**
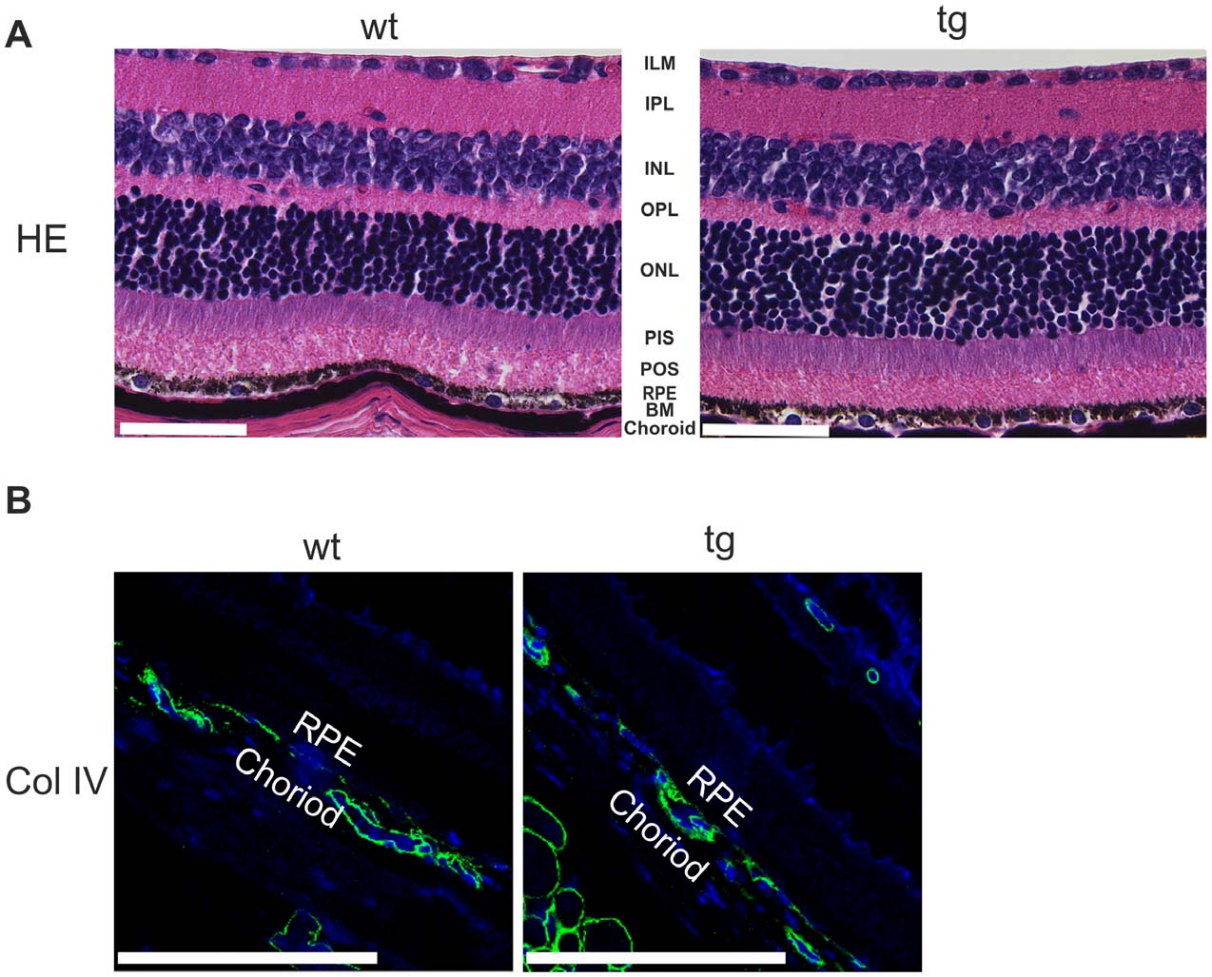
Paraffin cross sections of 3 month old WT and *Htra1* transgenic mice. (A) HE staining shown in original 40× magnification (Scale Bar: 100 µm). Mice show normal choroidal and retinal architecture (1mice/group, 3 independent experiments). Image is representative. ILM, inner limiting membrane; IPL, inner plexiform layer; INL, inner nuclear layer; OPL, outer plexiform layer; ONL, outer nuclear layer; PIS, photoreceptor inner segments; POS, photoreceptor outer segments; RPE, retinal pigment epithelium; BM, Bruch's membrane. (B) Immunofluorescence for collagen IV (Col IV) shown in original 100× magnification (Scale Bar: 100 µm). Transgenic mice show normally developed choroidal vasculature (1mice/group, 3 independent experiments). Image is representative. Green: Collagen IV, Blue: DAPI; RPE, retinal pigment epithelium.

Morphological screening of WT C57BL/6N and *Htra1* transgenic mice up to one year of age via hematoxylin-eosin (HE) staining on paraffin cross sections revealed no differences in choroid, BM and RPE (Fig. 2A). In addition, we observed no differences in vasculature by immunfluorescence for the endothelial cell marker Type-IV collagen (ColIV) (Fig. 2B).

### Ultrastructural changes in the elastic layer of Bruch's membrane in *Htra1* transgenic mice

**Figure 3:**
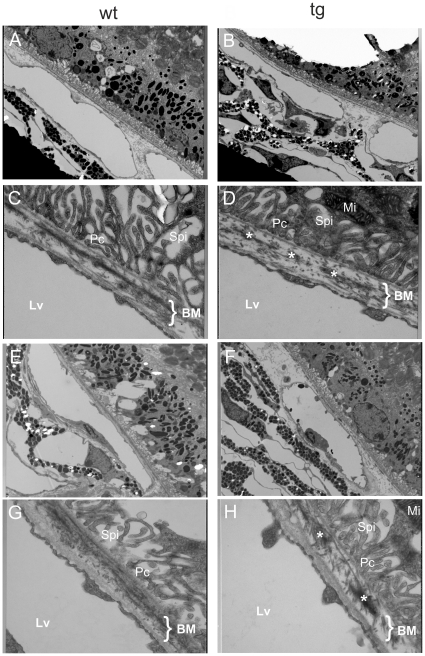
Transmission electron micrographs of BM. TEM images showing the elastic lamina of BM in WT (A, C, E, G) and *Htra1* transgenic (B, D, F, H) mice (A–D at the age of 3 month; E–H at the age of 12 month). A, B and E, F at original 3000× magnification and C, D and G, H at original 20000× magnification. Note the fragmentation of the elastic lamina in *Htra1* transgenic mice seen as discontinuities in contrast to the continuous elastic lamina of the WT mice. These discontinuities were observed in all transgenic mice that were analyzed (1mice/group, 6 independent experiments). Image is representative. BM, Bruch's membrane; Lv, Lumen vasculare (Lumen of a vessel); Mi, Mitochondria; Pc, Processus cellularis (cell process); Spi, Spatium intermembranosum (intermembranous cleft).

To further investigate the structural differences, we performed transmission electron microscopy (TEM). Here, *Htra1* transgenic mice revealed ultrastructural changes in the elastic layer of BM up to one year of age (Fig. 3 B, D, F and H). The elastic layer of *Htra1* transgenic mice appeared fragmented with spots of electron dense material (asterisk Fig. 3 D and H), while the elastic layer of control mice appeared thick and continuous (Fig. 3 A, C, E, G). To ensure that the discontinuities are not merely localized irregularities, the TEM images were made at the central retina as well as the periphery and graded through blinded and randomized examination by several independent graders. Fig. S2 indicates the widely spread morphological changes in the *Htra1* transgenic mice compared to WT.

### Expression of ECM proteins in RPE/choroid lysates of WT and *Htra1* transgenic mice

**Figure 4:**
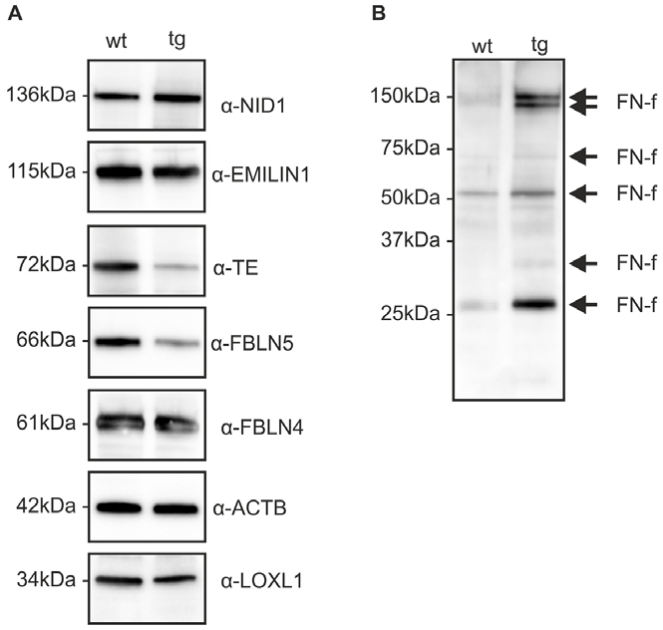
Western Blot analysis of RPE/choroid Lysates from WT (wt) and transgenic (tg) mice. (A) Transgenic mice show a lower expression of fibulin 5 (FBLN5) and tropoelastin (TE) compared to WT. Other ECM components like nidogen 1 (NID-1), EMILIN1, fibulin 4 (FBLN4) and LOXL1 are equally expressed (1mice/group, 6 independent experiments). Expression of ACTB serves as a loading control. Image is representative. (B) Transgenic mice demonstrate degradation of fibronectin with the generation of fibronectin fragments (FN-f) (1mice/group, 6 independent experiments). Image is representative.

Due to the changes observed in BM of *Htra1* transgenic mice in TEM, we conducted Western Blot analysis of RPE/choroid lysates to investigate the expression of major components of the ECM as well as the expression of ECM proteins that are involved in elastogenesis in *Htra1* transgenic compared to WT mice. Figure 4A clearly demonstrates that the expression of nidogen 1, elastin microfibril interface-located protein (EMILIN1), fibulin 4 and lysyl oxidase-like 1 (LOXL1) was comparable in WT and *Htra1* transgenic mice. Interestingly, expression levels of fibulin 5, which has elastogenic activity and tropoelastin were reduced in RPE/choroid lysates of *Htra1* transgenic mice compared to WT. Densitometric analysis of our Western Blot findings confirmed the reduction of fibulin 5 and tropoelastin levels (Fig. S3). Western Blot analysis also revealed higher expression of fibronectin fragments in transgenic mice (4B). These findings support the notion of an altered ECM homeostasis in *Htra1* transgenic mice.

### Distribution of nidogen 1 and nidogen 2 in BM

**Figure 5:**
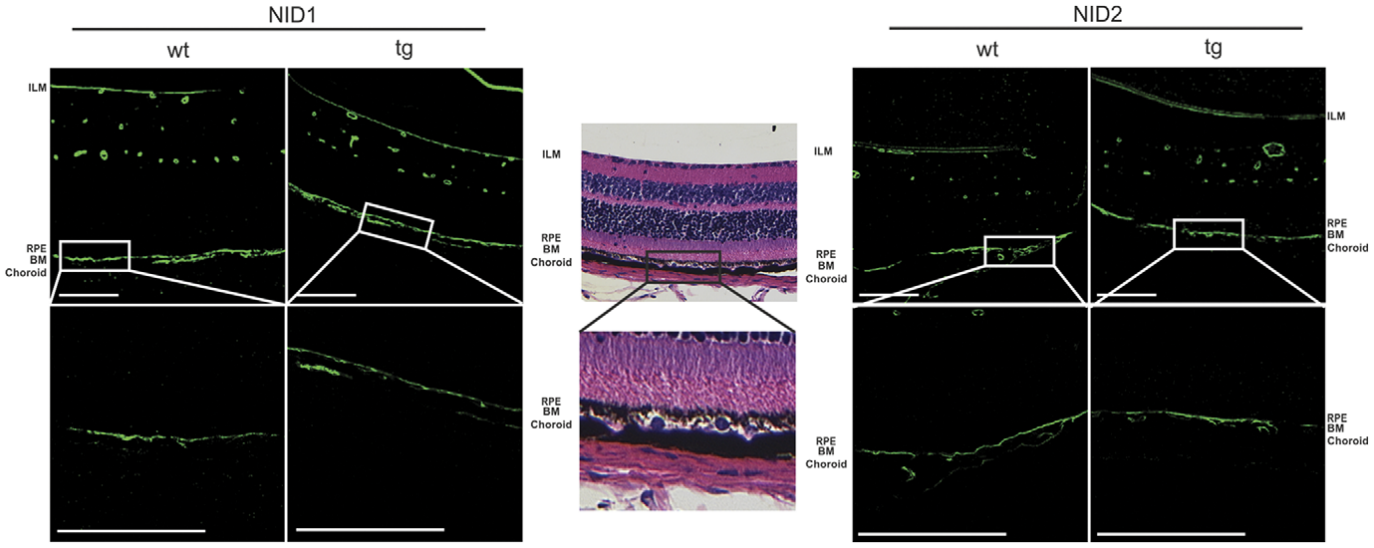
Immunostaining of ECM proteins in BM. (A) Immunofluorescence for nidogen 1 (NID1) and nidogen 2 (NID2) shown in original 40× and 100× magnification (Scale Bar: 100 µm). There is no difference in staining intensity observed between WT and transgenic mice (1mice/group, 3 independent experiments). Image is representative. Green∶nidogen 1 and nidogen 2, respectively. BM: Bruch's membrane; ILM: inner limiting membrane; RPE: retinal pigment epithelium. Image of H&E stained sections in 40× and 100× magnification provides orientation.

We performed immunofluorescence staining on paraffin sections of *Htra1* transgenic and WT mice to evaluate the presence of nidogen 1 and nidogen 2 in BM. Even though we detected degradation of nidogen 1 and nidogen 2 in our *in vitro* digestion assay, we observed no differences in the distribution of nidogen 1 and nidogen 2 in BM of WT and transgenic mice (Fig. 5).

### Proteolysis of ECM proteins by HTRA1

To analyze the effects of HTRA1 proteolytic activity on ECM components of BM, we purified recombinant human His-tagged HTRA1 lacking the N-terminal IGFBP/MAC25 domain (Δmac25HTRA1 plasmid kindly provided by Prof. Dr. Ehrmann, Duisburg-Essen, Germany). To ensure that the bands seen on Coomassie-stained SDS-Gel for the HTRA1 recombinant protein were indeed HTRA1 protein, we excised the two bands and processed them for mass spectrometry. The data showed that the two bands corresponded to human HTRA1 (Fig. S4). Proteolytic activity of recombinant HTRA1 was confirmed by ß-casein digestion (Fig. S5). An Inhibitor of HTRA1 (NVP-LEB748 kindly provided by Novartis) was used as a control to ensure that degradation of proteins was specifically due to HTRA1 proteolysis and not due to any possibly co-purified bacterial proteases. It was demonstrated to inhibit proteolytic activity by a dose-dependent manner with an IC_50_ of 0.21 µm as determined by HTRA1-dependent digestion of resorufin-labelled casein in an earlier study by Grau et al. [15].

**Figure 6:**
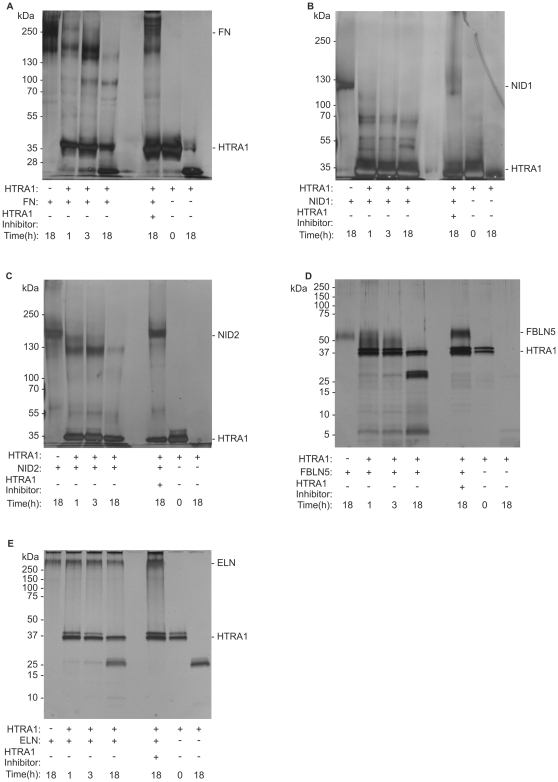
Degradation of ECM components by recombinant HTRA1. Purified ECM proteins and purified recombinant HTRA1 were incubated at 37°C in 50 mM Tris-HCl pH7,6, 5 mM CaCl_2_, 150 mM NaCl over a period of 18 hours (h). (A) Degradation of fibronectin. Fibronectin (FN) shows no sign of degradation when incubated alone. Recombinant HTRA1 degrades fibronection after three hours of incubation. The inhibitor of HTRA1 completely abolishes degradation of fibronectin by HTRA1. (B) Degradation of nidogen 1. Nidogen 1 (NID1) shows no sign of degradation when incubated alone. Recombinant HTRA1 cleaves nidogen-1 after one hours of incubation. The inhibitor of HTRA1 completely abolishes degradation of nidogen 1 by HtrA1. (C) Degradation of nidogen 2. Nidogen 2 (NID2) shows no sign of degradation when incubated alone. Recombinant HTRA1 cleaves nidogen 2 after one hours of incubation. The inhibitor of HTRA1 completely abolishes degradation of nidogen 2 by HTRA1. (D) Degradation of fibulin 5 by HTRA1. Fibulin 5 (FBLN5) is degraded by HTRA1 already after one hour, but shows no sign of degradation when incubated alone. The inhibitor of HTRA1 completely abolishes degradation of fibulin 5 by HTRA1. (E) Degradation of elastin. Elastin (ELN) shows no sign of degradation when incubated alone and when incubated with recombinant HTRA1.

Protease assays with purified ECM components were performed to screen for degradation of ECM components. As formerly described [15], fibronectin is a substrate of HTRA1 and was degraded after three hours of incubation with recombinant HTRA1 (Fig. 6A).

Nidogen 1 and nidogen 2, structural constituents of basement membranes, were also degraded by HTRA1 as shown in Fig. 6B and 6C. In contrast, laminin and Type-IV collagen showed no signs of degradation by recombinant HTRA1 *in vitro* (data not shown). Note that recombinant HTRA1 also degraded recombinant fibulin 5 (Fig. 6D). When using insoluble elastin from human skin as a substrate for HTRA1, we failed to observe any fragmentation bands for elastin (6E). Therefore, HTRA1 seemed to have no elastase activity.

After 18 hours of incubation and in the absence of substrate a loss of HTRA1 could be observed in all Figures. This loss was most likely due to self-degradation of HTRA1 as has been described before for bacterial HtrA [24] and human HTRA1 [25].

### TGF-ß expression in *Htra1* transgenic mice

**Figure 7:**
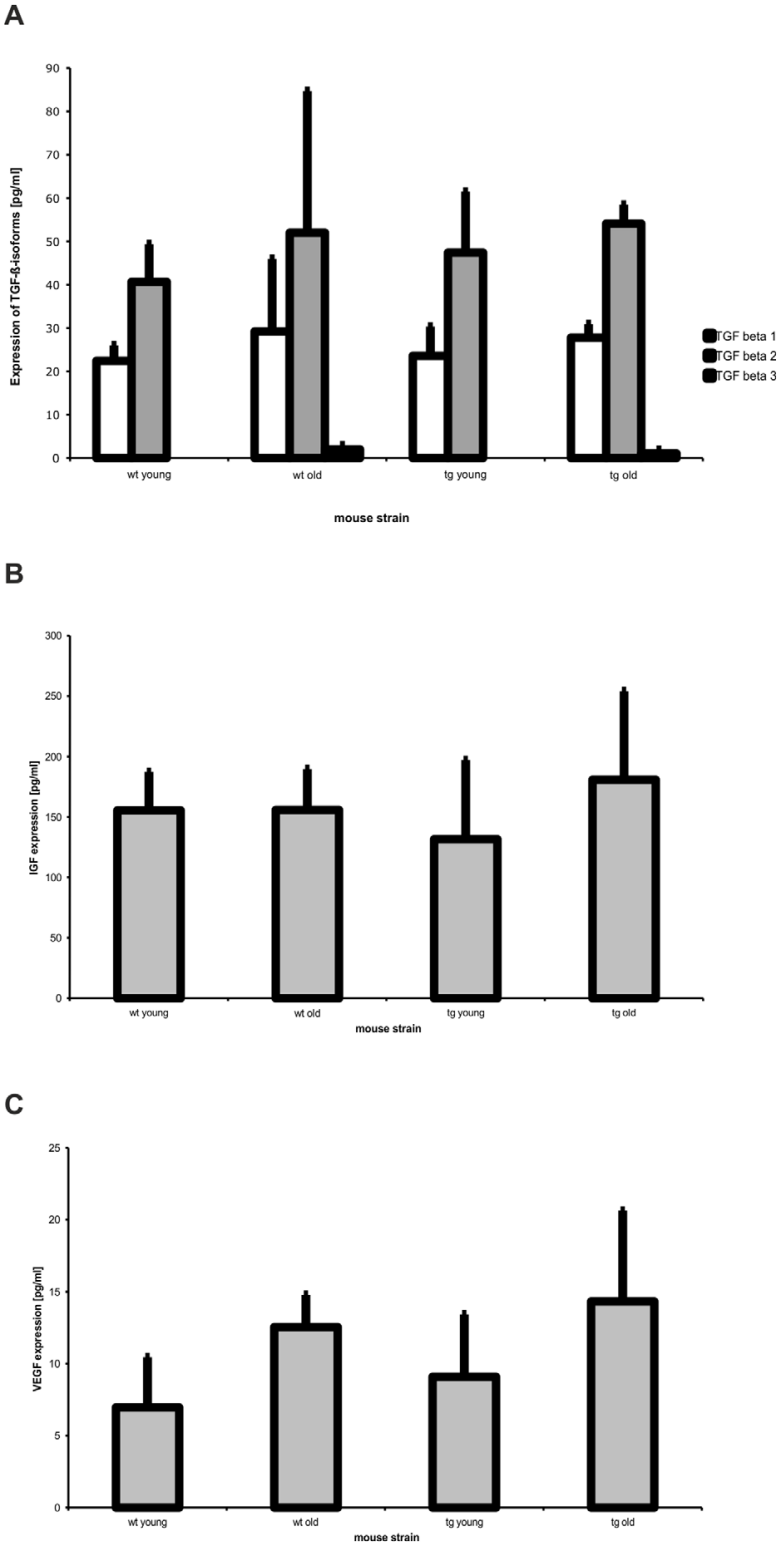
Luminex Assay of growth factors in RPE/choroid lysates. Concentration of growth factors in RPE/Choroid lysates of transgenic (tg) and WT (wt) mice at 3 month (young) and 12 month (old) of age (4mice/group, 3independent experiments). Data are presented as means; bars, ± SD. (A) Concentration of TGF-ß isoforms in RPE/choroid lysates. WT and transgenic mice show no significant differences in TGF-ß isoform concentration at young or old age. (B) Concentration of IGF-1 in RPE/choroid lysates. WT and transgenic mice show no significant differences in IGF-1 concentration at young or old age. (C) Concentration of VEGF in RPE/choroid lysates. WT and transgenic mice show no significant differences in VEGF concentration at young or old age.

Since HTRA1 is known to inhibit TGF-ß and has an IGF-binding protein (IGFBP) domain, we were interested in the TGF-ß and IGF-1 expression in the RPE/choroid layer of *Htra1* transgenic mice. We also determined VEGF expression levels because of its pro-angiogenic properties. To investigate this expression, we performed Multiplex assays via the Luminex xMAP Technology (Fig. 7A–C). Transgenic and WT mice showed no significant differences in the concentration of these growth factors at young or old age.

## Discussion

Polymorphisms in the chromosomal region 10q26 are highly associated with AMD. Two attractive candidate genes reside in this region: *ARMS2* and *HTRA1*. There is conflicting data concerning the involvement of these genes in the pathogenesis of AMD [9], [10], [11], [12], [13]. In this study, we provide the first functional evidence that HTRA1 overexpression in RPE is involved in AMD pathogenesis by fragmentation of the elastic layer in BM.

It has long been postulated that BM is important in AMD development and particularly in formation of choroidal neovascularisation (CNV) in exsudative AMD [26]. Since BM is an acellular layer, it depends on the adjacent RPE and choroidal cells for most of its ECM constituents [27]. With aging a loss of elastic fibers, thickening and calcifications of BM have been observed [28]. In this study, transgenic mice overexpressing HTRA1 showed ultrastructural changes of the elastic layer by TEM. Subsequent Western Blot analysis of major components of the ECM as well as ECM proteins involved in elastogenesis revealed an increase in fibronectin fragments and a reduction of fibulin 5 expression in *Htra1* transgenic mice compared to WT in RPE/choroid lysates. *In vitro* assays showed that HTRA1 degrades fibulin 5 as well as fibronectin. In addition, it also degraded nidogen 1 and nidogen 2, which are important structural constituents of basement membranes. Fibronectin fragments stimulate the release of cytokines and MMPs from RPE cells [29] and therefore can alter the homeostasis of the ECM, which can lead to further degradation of ECM components. Elevated levels of fibronectin fragments are often found in synovial fluids of patients with rheumatoid arthrithis [30] and thus are linked to inflammation. Moreover, intact fibronectin is better able to bind collagen and mediate its cell binding, thereby stabilizing the ECM. In contrast, fibronectin fragments have a decreased ability to form and stabilize the ECM.

While we could observe degradation of nidogen 1 and nidogen 2 *in vitro*, we failed to observe degradation of nidogen in *Htra1* transgenic mice. Our *in vitro* assays, however, might not reflect physiological conditions due to the fact that we used recombinant HTRA1 without the IGFBP/MAC25 homology domain, which contains an inhibitory element and therefore might have a higher protease activity than endogenous HTRA1.

Human recombinant HTRA1 failed to cleave insoluble elastin from human skin, thereby showing that the fragmentation of the elastic layer in *Htra1* transgenic mice is not due to an elastase activity of HtrA1.

Missense mutations of the fibulin 5 gene have been associated with AMD [31] and reduced secretion of fibulin 5 has been found in four AMD mutations [32]. Fibulin 5 is known to be essential for elastogenesis *in vivo* as observed with fibulin 5^−/−^ mice [33]. Additionally, we were able to demonstrate that transgenic mice also have lower tropoelastin expression in RPE lysates. Fibulin 5 binds to tropoelastin [34], [35] and accelerates self-aggregation of tropoelastin, a process called coacervation [36]. Together with the finding of a fragmented elastic lamina in *Htra1* transgenic mice, this result indicates an altered elastic fiber formation due to the reduction of fibulin 5. Moreover, our *in vitro* studies demonstrate that HTRA1 cleaves fibulin 5. In 2007, Hirai et al. [37] reported that the elastogenic organizer activity of fibulin 5 is abrogated by proteolytic cleavage, but failed to identify the responsible serine protease. Here, we report recombinant human HTRA1, a serine protease to cleave fibulin 5 *in vitro*. Fibulin 5 promotes elastic fiber assembly and maturation by organizing tropoelastin, LTBP-2 and the crosslinking lysyl oxidase-like enzymes LOXL1, 2 and 4 along Fibrillin microfibrils [38], [39], [40], [41]. Besides, Yu et al. [42] described fragmentation of the elastic laminar in mice lacking LOXL1. While we observed a reduction in fibulin 5 and tropoelastin expression, we didn't observe changes in the expression levels of other proteins that take part in elastogenesis. Therefore, it seems that the reduction of fibulin 5 and neither fibulin 4, nor EMILIN1, nor LOXL1 impairs elastogenesis in *Htra1* transgenic mice and is responsible for the fragmentation of the elastic layer in BM as seen in TEM.

**Figure 8:**
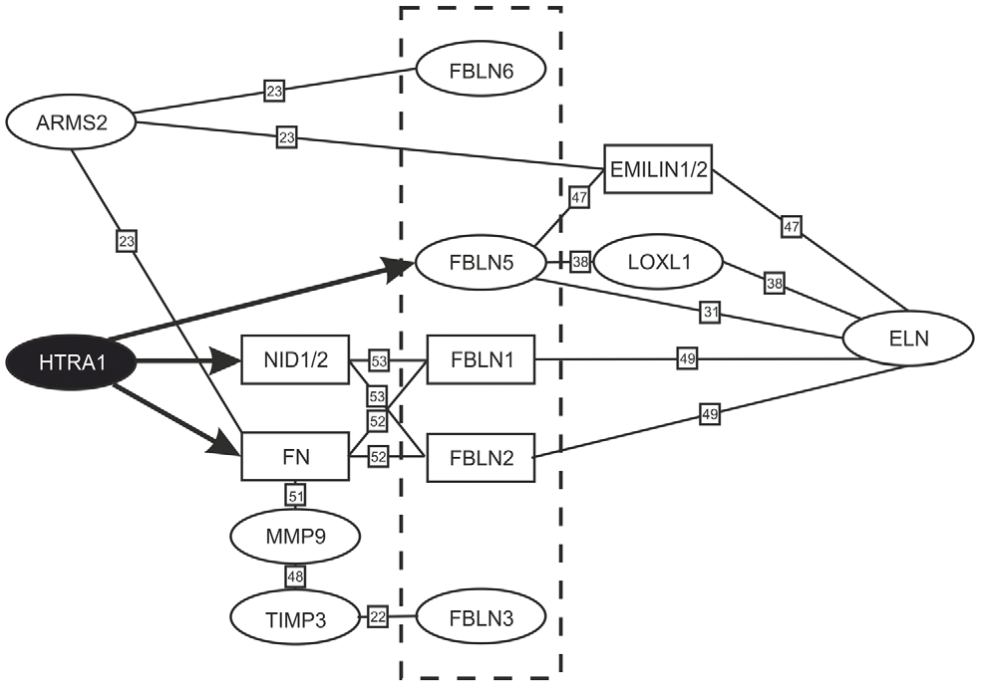
HTRA1 interacts with ECM proteins. Black arrows indicate cleavage by HTRA1. Proteins encoded by genes known to be related to macular degenerations are circled. Fibulins are delineated by the dashed box. Note that three of five fibulin genes shown have been implicated in maculopathies, and one of five directly interacts with HTRA1. Reference(s) for interactions described earlier are indicated in small boxes. [22], [23], [31], [38], [47]–[53] ARMS2, age-related macular susceptibility 2; ELN, elastin; EMILIN, elastin microfibril interface-located protein; FBLN, fibulin; FN, fibronectin; HTRA1, high-temperature requirement A1; LOXL1, lysyl oxidase-like 1; MMP9, matrix metalloproteinase-9; NID, Nidogen; TIMP3, tissue inhibitor of metalloproteinase 3.

This data links HTRA1 into a network of extracellular proteins involved in AMD (Fig. 8). In addition to fibulin 5, other fibulin proteins are implicated in macular dystrophies. Recently, it was shown that ARMS2 interacts with fibulin 6 causing a form of famililar AMD [23]. Elastic fibers in BM seem to be crucial in the pathogenesis in AMD. Both fibulins and EMILIN2 (elastin microfibril interface located protein), another interacting partner of ARMS2 are involved in elastin biogenesis. The importance of elastin is further supported by the fact that the concentration of serum elastin-derived peptides are higher in patients with AMD than in control subjects [43].

Angiogenesis, a prerequisite of CNV in AMD, is promoted by both TGF-ß and VEGF. HTRA1 has been demonstrated to inhibit TGF-ß1 signaling in neuronal cells [44] and is associated with cerebral autosomal recessive arteriopathy with subcortical infarcts and leukoencephalopathy (CARASIL) [19]. To test if HTRA1 inhibition of TGF-ß has any effect on the expression levels of TGF-ß in *Htra1* transgenic mice, we measured expression levels in a Luminex assay. There was no significant difference in expression levels for TGF-ß1, 2 and 3 in transgenic compared to WT mice. Thus, we conclude that the inhibition of TGF-ß by HTRA1 is negligible in RPE/choroid lysates of *Htra1* transgenic mice.

HTRA1 is also known to have an IGFBP/MAC25 homology domain and is capable of binding IGF-1. Our results exhibit no significant changes in IGF-1 and VEGF expression levels in *Htra1* transgenic mice and consequently angiogenesis is not differentially regulated in *Htra1* transgenic mice compared to WT mice regarding these factors.

In summary, our data suggests that an overexpression of HTRA1 in the RPE leads to an altered BM with fragmentation of the elastic laminar due to a decrease in fibulin 5, which could be shown by Western Blot analysis and degradation by recombinant HTRA1 *in vitro*. Furthermore, we demonstrate that HTRA1 degrades nidogen 1, nidogen 2 and fibronectin, but not elastin *in vitro*. Western Blot analysis also confirmed the degradation of fibronectin with a higher expression of active fibronectin fragments in *Htra1* transgenic mice. These fragments are known to have proteolytic activity [45] and stimulate the release of cytokines and MMPs, which might further contribute to destabilization of the ECM. This data highlights the importance of ECM related proteins in the development of AMD. It links *HTRA1* to other AMD risk genes such as fibulin 5, fibulin 6, *ARMS2* and *TIMP3*.

## Materials and Methods

### Ethics Statement

This study utilized experiments using mice. All experiments using mice were performed with consent from the Animal Experimentation Committee of the University of Cologne (9.93.2.10.31.07.045). The study did not involve human experiments.

### Generation of transgenic mice

**Figure 9:**
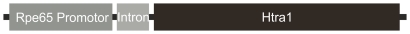
Scheme of the transgenic construct Rpe65/Htra1 (3123 bp).

A full-length *Htra1* cDNA (2030 bp), generated from mouse cDNA by PCR, was cloned directionally after the murine *Rpe*65 promoter into a pCI plasmid (Promega) (Fig. 9). The *Rpe*65 promotor confers tissue specific expression as described by Boulanger et al. [46]. The inclusion of a generic intron is used to further maximize expression. The sequence of the final construct was confirmed (3123 bp) by DNA sequencing. The construct was digested with PagI and Bsu15I followed by purification of the 3.9-kb fragment via agarose gel electrophoresis and millipore membrane filtration. The linearised transgene was used to generate seven founder mice via microinjection (ZMMK Service). The mice were screened for the integrity of the transgene by sequencing through the entire construct. To expand the transgenic lines, the founder mice were crossed into a C57BL/6N background (Charles River Laboratories). All animals investigated were derived from founder no. 2 and were heterozygous for the transgene, as they were generated by mating a transgenic parent with a C57BL/6N mouse.

### PCR

Mice were screened for the presence of the transgene by PCR on genomic DNA. Tail pieces were digested overnight at 56°C in lysis buffer (High Pure PCR Template Preparation Kit, Roche), supplemented with 20 mg/ml proteinase K. DNA was isolated and quantified according to the manufacturer's instructions. For the amplification of the transgene-specific sequence (417 bp) at 56°C, a 5′-primer (5′- GCAGGCTCCTCCAATAAGG -3′) and a 3′-primer (5′- AAGGAAGGAGCCGCTAGCAG -3′) were used to produce a 417 bp product. The amplicons were separated by agarose gel electrophoresis and detected by ethidium bromide staining.

### Real-time PCR

Total RNA was isolated from RPE/Choroid layers of transgenic and WT mice, at the age of 3 month, using the RNeasy Mini Kit (QIAGEN). The cDNA was synthesized with oligo (dT) Primer using the SuperScript III First-Strand Synthesis SuperMix (Invitrogen) followed by a RNase H digestion step.

PCR was performed using 12,5 µl of Platinum qPCR SuperMix-UGD (Invitrogen), 0,25 µl Universal Probe (Roche), labeled at the 5′ end with fluorescein (FAM) and at the 3′ end with a dark quencher dye, PCR Primers and 5 µl cDNA. Real-time PCR was performed using the MP 3000 detection system (Stratagene) and the PCR reaction was carried out at 50°C for 2 minutes and 95°C for 2 min followed by 50 cycles of 95°C for 15 seconds and 60°C for 30 seconds. At the end of the PCR cycles, a melting curve, using a temperature range from 55°C to 95°C with +0.5°C intervals, was generated to test the specificity of the PCR product. A cDNA sample in each experiment was tested in triplicate and each experiment was performed twice. The forward primer for *Htra1* was 5′- AGTGGGTCAGGATTCATCGTA -3′ and the reverse primer was 5′- GTGACCACGTGAGCATTTGT -3′. As Universal Probe for HtrA1 PCR we selected Universal Probe No. 88. *Actb* served as the internal control. The forward primer for *Actb* was 5′- CCTCACCCTCCCAAAAGC -3′ and the reverse primer was 5′- GTGGACTCAGGGCATGGA -3′. As Universal Probe for *Actb* PCR we selected Universal Probe No. 101. PCR efficiency represented by a standard curve reached over 95%.

### Cell culture

Primary murine RPE cells were isolated from freshly dissected eyes and cultured in medium Dulbecco's modified Eagle's medium (high glucose, w/glutamine; Gibco Invitrogen) supplemented with 10% fetal bovine serum (PAA), and antibiotics (100 Units/ml penicillin and 100 µg/ml streptomycin; PAA. Cells were cultured till confluent and medium was exchanged by fetal serum serum-free medium 24 hours prior to Western Blot experiments.

### Western blot analysis of murine RPE/choroid Lysates

The expression of HTRA1, fibulin 5, fibulin 4, nidogen 1, EMILIN1, LOXL1 and tropoelastin in the RPE layer of transgenic and WT mice was evaluated by immunoblotting. Briefly, the vitreous and retina were removed and RPE/choroid layers were lysed for 30 min on ice in lysis buffer (RIPA, Bio-Rad Laboratories) supplemented with a mixture of protease inhibitors (Bio-Rad Laboratories). The samples were cleared by a centrifugation step (30 min at 10000 g) and the supernatant was assayed for protein concentration (BCA assay, Thermo Fisher Scientific). Protein (10 µg) was electrophoresed on AnyKD TGX gel (Bio-Rad Laboratories). Proteins were electrophoretically transferred to a PVDF membrane (GE Healthcare) and then blocked in 5% non-fat milk. After washing blots were probed with antibodies to HTRA1 (1∶1000, rabbit polyclonal, generated from recombinant HTRA1 lacking the Mac25 homology domain by Pineda), fibulin 5 (1∶400, rabbit polyclonal, Santa Cruz Biotechnology), tropoelastin (1∶1000, rabbit polyclonal, PR385 Elastin Products Company), fibulin 4 (1∶1000, rabbit polyclonal, Santa Cruz Biotechnology), EMILIN1 (1∶1000, rabbit polyclonal, Santa Cruz Biotechnology), LOXL1 (1∶200, rabbit polyclonal, Santa Cruz Biotechnology), ACTB (1∶10000, mouse monoclonal, Abcam). Polyclonal antibodies against nidogen 1 and fibronectin were kindly provided by Ursula Hartmann (Köln, Germany) and were used in a 1∶1000 dilution. After washing, the respective secondary peroxidase-labeled antibody (Dako) was applied at a 1∶4000 dilution for 1 h at room temperature. Immunoreactive detection was by chemiluminescence (ECL Plus Western Blotting Chemiluminescent Substrate, GE Healthcare). Subsequent densitometric analysis was performed using the Quantity One Software (Bio-Rad Laboratories). To account for variations in the applied quantity of protein, the analysis was made for the signal intensity of HTRA1 as well as for the signal intensity of the loading control. The relative signal intensity was calculated by dividing the signal intensity of HTRA1 with the signal intensity of ACTB.

### Immunofluorescence

Eyes from 3-month-old C57BL/6N and *Htra1* transgenic mice were enucleated, fixed overnight in methacarn (60% methanol, 30% 1,1,1-trichloroethane and 10% acetic acid), dehydrated and embedded in paraffin. Sagittal cross-sections were cut at 5 µm, deparaffinized and hydrated. After treatment for 10 min with 0.005% trypsin at 37°C, sections were blocked for 1 h at room temperature with 5% BSA and 0.1% Triton X-100 in 0.15 M NaCl and 50 m M Tris-HCl at pH 7.4 (TBS). They were then incubated overnight at 4°C with a primary antibody, followed by 1-hour incubation at room temperature with a fluorochrome-labeled secondary antibody (antirabbit Alexa 488; Invitrogen) in 1∶1000 dilution. Slides were examined under a Leica TCS SP5 sectral confocal microscope.

### Transmission Electron Microscopy

For morphologic analysis, eyes were removed and then processed for transmission electron microscopy by conventional techniques. Tissue was placed in fixative consisting of 2.5% glutaraldehyde and 2% formaldehyde in 0.1 M cacodylate buffer and was fixed for 12 to 24 hours at 4°C. The specimens were subsequently changed to 0.1 M cacodylate buffer for storage at 4°C. The tissue was trimmed to block size and dehydrated in ethanol prior infiltration with mixtures of propylene oxide and Epon 812 (Fluka), embedded in pure Epon 812, and polymerized at 60°C for 18 to 24 hours. One-micrometer sections and thin sections were cut on an ultramicrotome (Ultracut UCT, Leica). The 1 µm sections were stained with 0.5% toluidine blue and the thin sections were stained with saturated aqueous uranyl acetate and lead stain and then examined under a transmission electron microscope (EM 902A, Zeiss).

### Expression and Purification of HTRA1

The pETΔmac25HTRA1 plasmid was kindly provided by Michael Ehrmann (Essen-Duisburg, Germany). E. coli strain DE3^+^ was transformed with the plasmid and grown at 37°C while shaking till it reached an OD_600_ of 0.5. Protein expression was induced with 0.5 mM IPTG. After 5 hours at 25°C the cells were centrifuged (30 min at 4000 g) and the pellet was resuspended in lysis buffer (500 mM NaH_2_PO_4_, 3 M NaCl, pH 7.5) followed by homogenization and centrifugation (30 min at 15000 rpm) Precleared cell lysate was loaded on a Ni^2+^-NTA Superflow column (QIAGEN) at a flow rate of 3 ml/min. The column was washed with wash buffer I (100 mM Tris, 150 mM NaCl, pH 7.5) and buffer II (100 mM Tris, 150 mM NaCl, pH 7.5, 5 mM ß-Mercaptoethanol, 30 mM imidazole) prior to elution in elution buffer (100 mM Tris, 150 mM NaCl, pH 7.5, 5 mM ß-Mercaptoethanol, 150 mM imidazole). Fractions of 1 ml were collected and analyzed by SDS-PAGE. The purest fractions were then loaded to a HPS column (Bio-Rad Laboratories) at a flow rate of 3 ml/min. The column was washed with buffer I (50 mM NaCl, 50 mM HEPES/NaOH, adjust pH to buffer II) and the protein was eluted with buffer II (50 mM NaCl, 50 mM HEPES/NaOH, 500 mM KH_2_PO_4_). Fractions of 4 ml were collected and analyzed by SDS-PAGE. Protein bands were then cut from the gel and analyzed via peptide mass fingerprinting (PMF) by the bioanalytic department of the Center for Molecular Medicine, University of Cologne (CMMC) to confirm that the purified protein is recombinant HTRA1. Purified protein was stored in aliquots in storage buffer (50 mM Tris-HCl, 500 mM NaCl, pH 8.0, 10% glycerol) at −70°C.

### Digestion Assays

Degradation of fibronectin (Sigma-Aldrich), fibulin 5 (R&D Systems), nidogen 1, nidogen 2 (kindly provided by Manuel Koch, Köln, Germany) and insoluble elastin from human skin (Sigma-Aldrich) was determined by incubation of recombinant HTRA1 with these proteins in 50 mM Tris-HCl, pH 8.5, 150 mM NaCl for 1, 3 or 18 h at 37°C. ECM proteins and HTRA1 were incubated separately under the same conditions and served as controls. HTRA1 inhibitor (NVP-LEB748 kindly provided by Novartis) was preincubated with HTRA1 for 20 min at room temperature at a final concentration of 5 µM prior to the addition of ECM substrates. Samples were analyzed on a Silver-stained gel.

### Luminex Assays

For all Assays, MILLIPLEX Map Kits from Millipore were used. RPE/Choroid lysates, standards, and quality control samples (25 µl) were added to a pre-wet filter plate in 96-well format followed by addition of premixed microspheres. After an overnight incubation (protected from light; continuous shaking), at 4°C a detection antibodies were added to each well (25 µl) and were incubated for 1 hour at room temperature. The antigen–antibody complex was detected by a phycoerythrin-labeled streptavidin conjugate (25 µl) that was incubated for 30 min at room temperature. Following a wash step, the signal associated with each analyte was measured by analyzing the resulting bead-capture antibody-analyte-detection antibody-Streptavidin-Phycoerythrin conjugate complexes on the Luminex 200. The analyte-specific capture antibodies covalently coupled to spectrally unique microspheric beads provide analytical specificity. The analyte concentration is related to the fluorescence intensity derived from complexed detection conjugates.

## Supporting Information

Figure S1 **Expression levels of HTRA1 shown by Western Blot and subsequent densitometric analysis.** (A) Western Blot analysis of RPE/choroid lysates from 3month old WT (wt) and transgenic (tg) mice. Transgenic mice show a higher expression of HTRA1 protein compared to WT. Expression of ACTB serves as a loading control. (B) Densitometric analysis of HTRA1 expression. The graph demonstrates the relative signal intensity of WT (wt) and transgenic (tg) mice. Htra1 transgenic mice show a 2.68 fold increase in signal intensity compared to WT mice.
(TIF)

Figure S2 **Transmission electron micrographs of BM.** TEM images showing the elastic lamina of BM in WT (wt) and *Htra1* transgenic (tg) mice at the age of 3 month and at the age of 12 month at original 3000× magnification. Note that the discontinuities are no local irregularities.
(TIF)

Figure S3 **Expression levels of ECM proteins shown by Western Blot and subsequent densitometric analysis.** (A) Western Blot analysis of RPE/choroid lysates from 3month old WT (wt) and transgenic (tg) mice. Transgenic mice demonstrate moderate differences in expression of nidogen 1 (NID1), elastin microfibril interface-located protein (EMILIN1), fibulin 4 (FBLN4) and lysyl oxidase-like 1 (LOXL1), protein compared to WT. In contrast, fibulin 5 (FBLN5) and tropoelastin (TE) show high reduction of expression levels. Expression of ACTB serves as a loading control. (B) Densitometric analysis of ECM protein expression. The graph demonstrates the relative signal intensity of WT (wt) and transgenic (tg) mice.
(TIF)

Figure S4 **Quality control of human recombinant Δmac25HTRA1.** Human recombinant HTRA1 protein showed two bands when incubated alone and analyzed by SDS-PAGE (left panel). To ensure the quality of recombinant HTRA1 and rule out potential contamination with other proteases band (A) and (B) were excised and analyzed by PMF as described in the method. Right panel shows the corresponding HTRA1 tryptic peptides detected in each band.
(TIF)

Figure S5 **Degradation of ß-casein by recombinant HTRA1.** Purified ß-casein and purified recombinant HTRA1 were incubated at 37°C in 50 mM Tris-HCl pH7,6, 5 mM CaCl2, 150 mM NaCl over a period of 3 hours (h). There are no signs of ß-casein degradation when incubated alone. Recombinant HTRA1 degrades ß-casein already after 0.5 hours. The inhibitor of HTRA1 completely abolishes degradation of ß-casein by HTRA1.
(TIF)
