# Supplementary figures and images for: Overexpression of HTRA1 Leads to Ultrastructural Changes in the Elastic Layer of Bruch's Membrane via Cleavage of Extracellular Matrix Components
Source: PLoS One. 2011 Aug 2;6(8):e22959. doi: 10.1371/journal.pone.0022959 (PMC3149070; doi:10.1371/journal.pone.0022959)

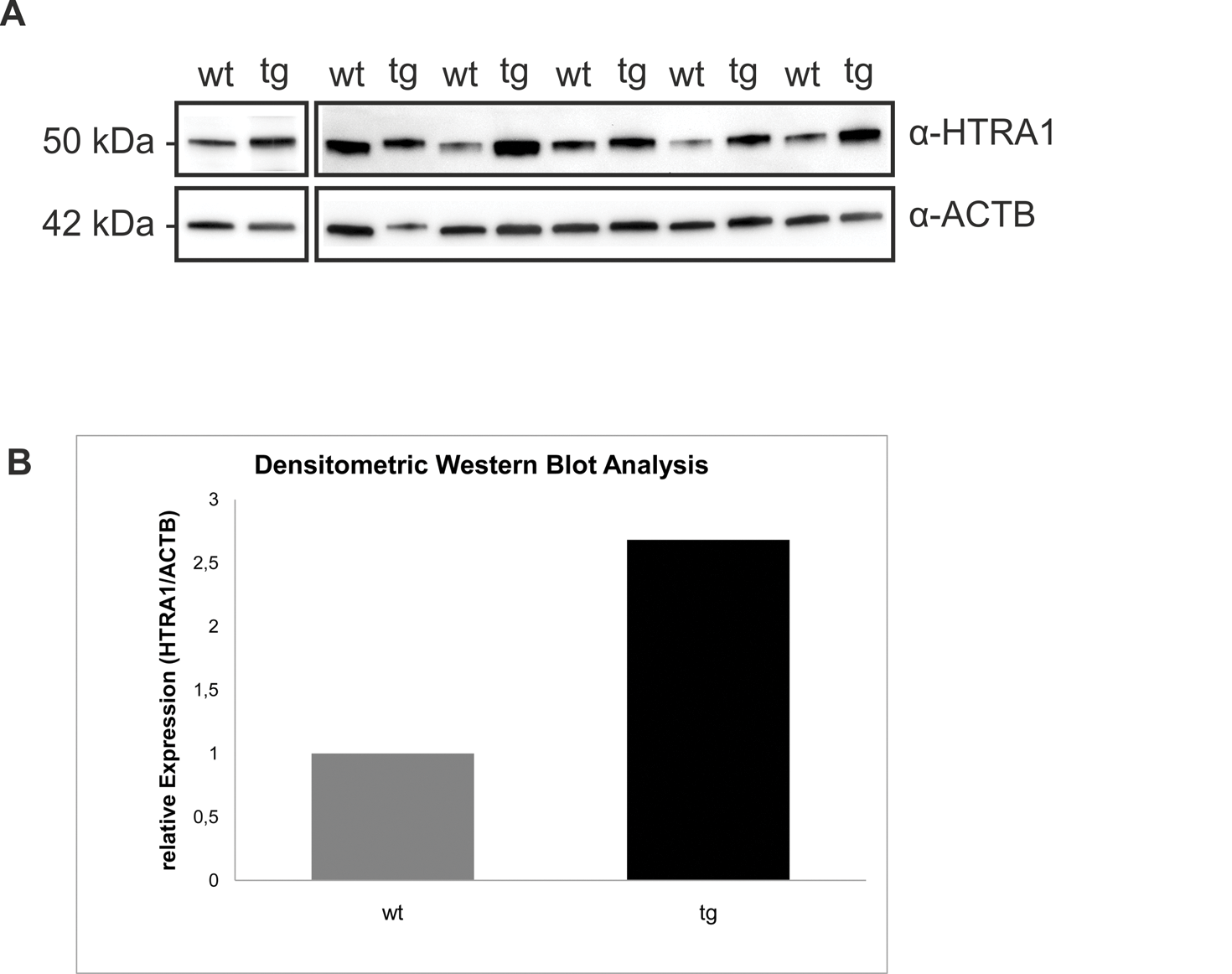

Supplement: Figure S1 — Expression levels of HTRA1 shown by Western Blot and subsequent densitometric analysis. (A) Western Blot analysis of RPE/choroid lysates from 3month old WT (wt) and transgenic (tg) mice. Transgenic mice show a higher expression of HTRA1 protein compared to WT. Expression of ACTB serves as a loading control. (B) Densitometric analysis of HTRA1 expression. The graph demonstrates the relative signal intensity of WT (wt) and transgenic (tg) mice. Htra1 transgenic mice show a 2.68 fold increase in signal intensity compared to WT mice. (TIF) [file pone.0022959.s001.tif]

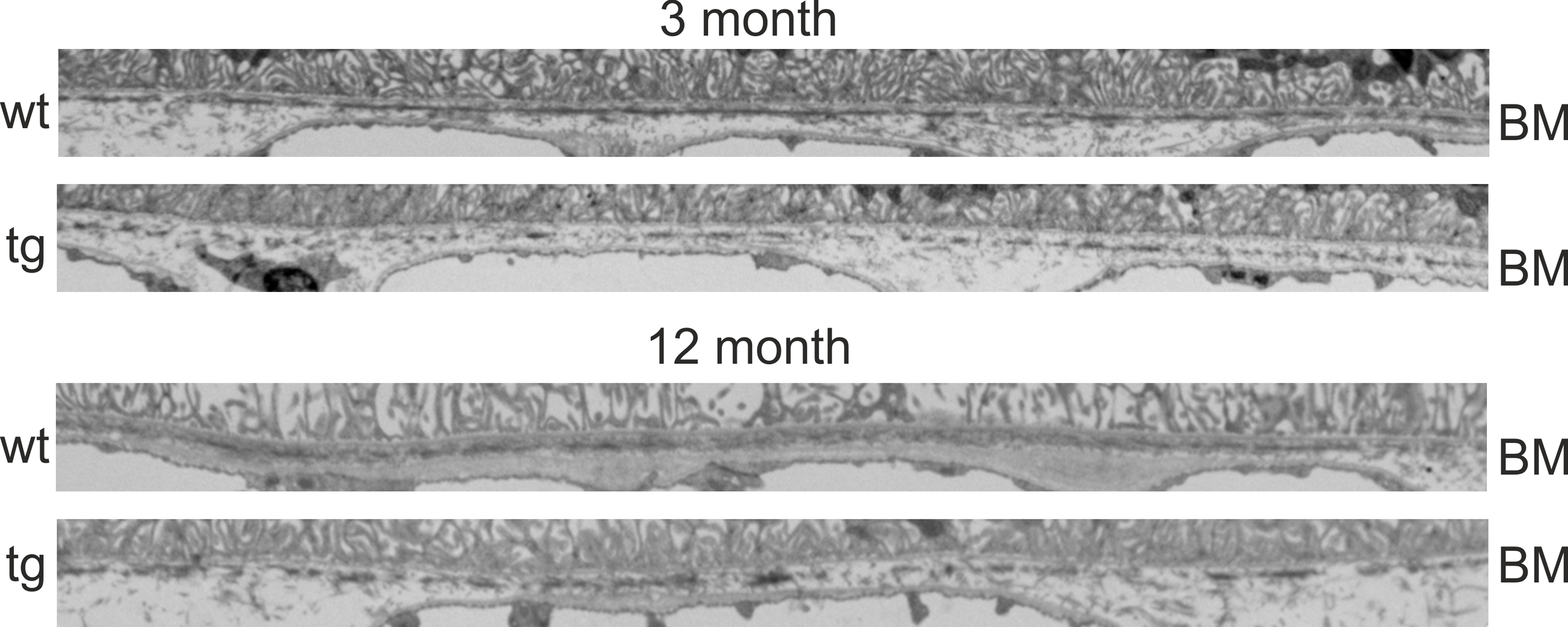

Supplement: Figure S2 — Transmission electron micrographs of BM. TEM images showing the elastic lamina of BM in WT (wt) and Htra1 transgenic (tg) mice at the age of 3 month and at the age of 12 month at original 3000× magnification. Note that the discontinuities are no local irregularities. (TIF) [file pone.0022959.s002.tif]

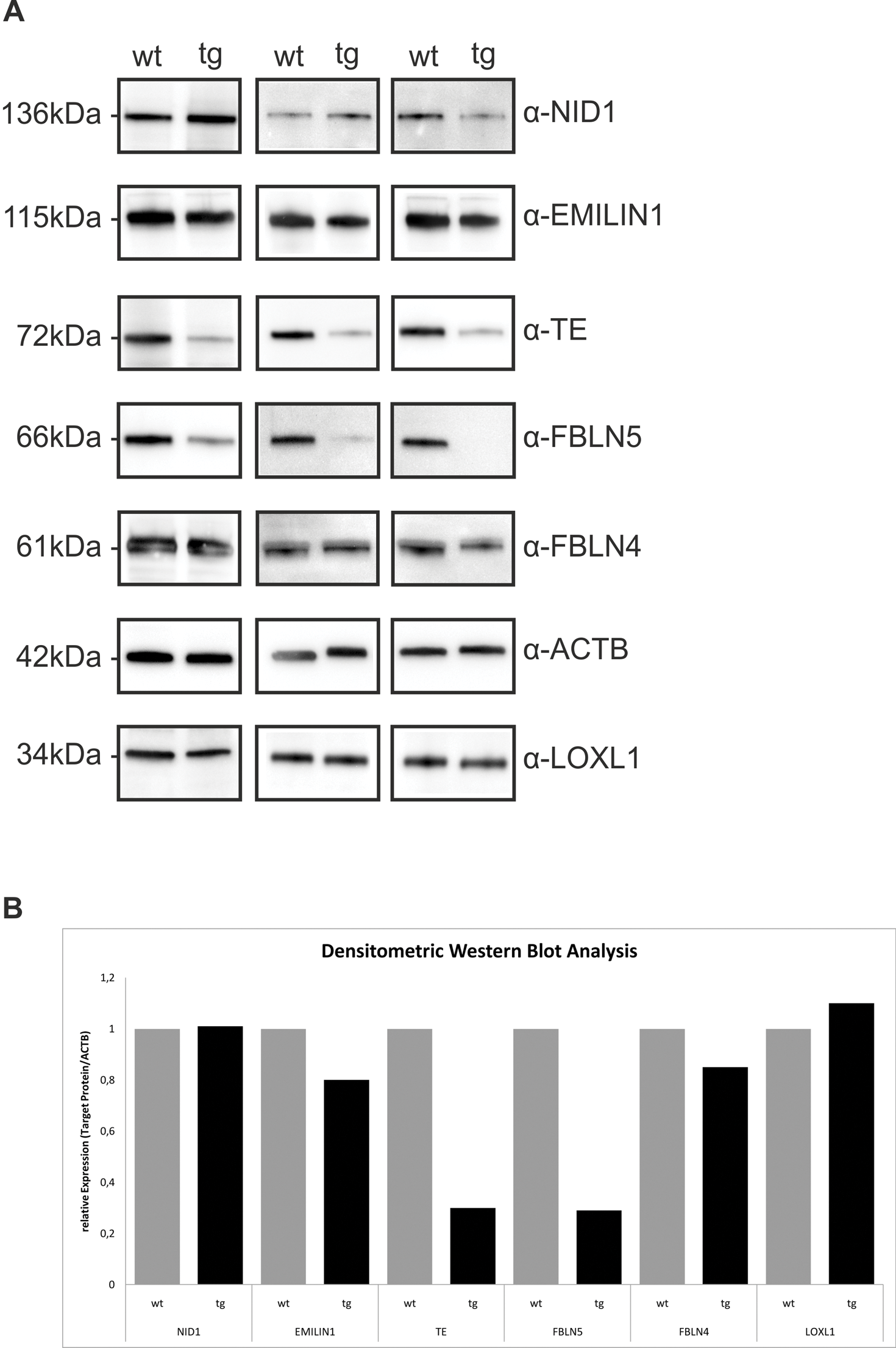

Supplement: Figure S3 — Expression levels of ECM proteins shown by Western Blot and subsequent densitometric analysis. (A) Western Blot analysis of RPE/choroid lysates from 3month old WT (wt) and transgenic (tg) mice. Transgenic mice demonstrate moderate differences in expression of nidogen 1 (NID1), elastin microfibril interface-located protein (EMILIN1), fibulin 4 (FBLN4) and lysyl oxidase-like 1 (LOXL1), protein compared to WT. In contrast, fibulin 5 (FBLN5) and tropoelastin (TE) show high reduction of expression levels. Expression of ACTB serves as a loading control. (B) Densitometric analysis of ECM protein expression. The graph demonstrates the relative signal intensity of WT (wt) and transgenic (tg) mice. (TIF) [file pone.0022959.s003.tif]

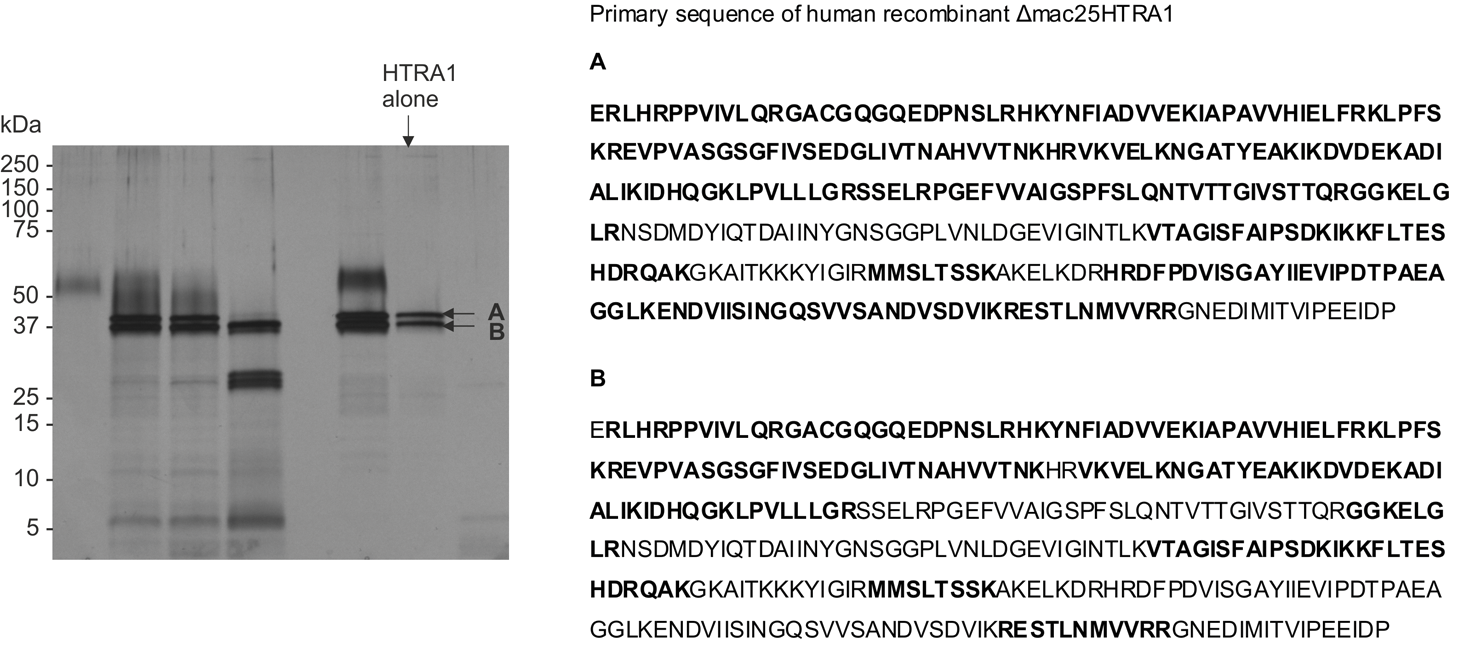

Supplement: Figure S4 — Quality control of human recombinant Δmac25HTRA1. Human recombinant HTRA1 protein showed two bands when incubated alone and analyzed by SDS-PAGE (left panel). To ensure the quality of recombinant HTRA1 and rule out potential contamination with other proteases band (A) and (B) were excised and analyzed by PMF as described in the method. Right panel shows the corresponding HTRA1 tryptic peptides detected in each band. (TIF) [file pone.0022959.s004.tif]

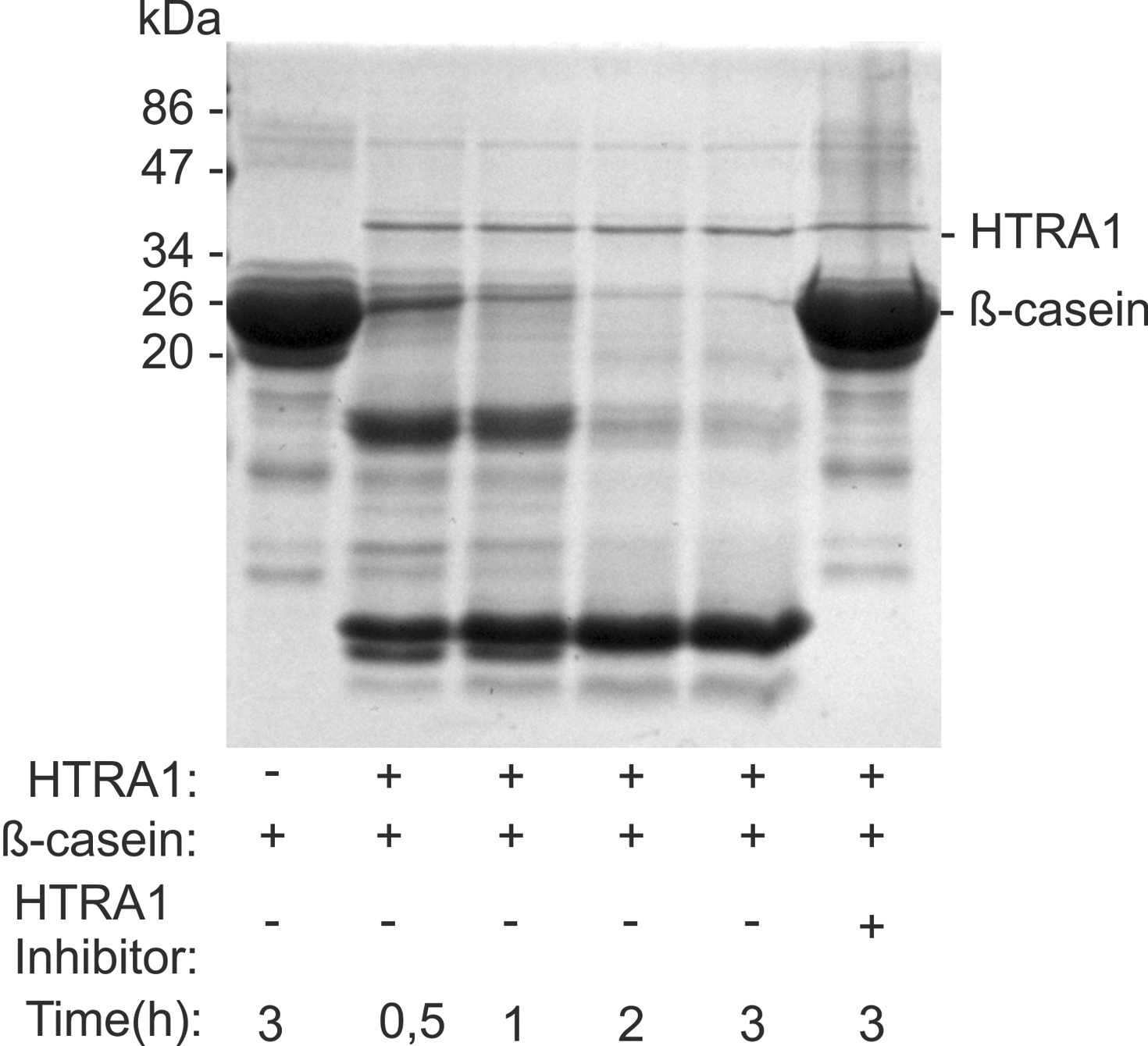

Supplement: Figure S5 — Degradation of ß-casein by recombinant HTRA1. Purified ß-casein and purified recombinant HTRA1 were incubated at 37°C in 50 mM Tris-HCl pH7,6, 5 mM CaCl2, 150 mM NaCl over a period of 3 hours (h). There are no signs of ß-casein degradation when incubated alone. Recombinant HTRA1 degrades ß-casein already after 0.5 hours. The inhibitor of HTRA1 completely abolishes degradation of ß-casein by HTRA1. (TIF) [file pone.0022959.s005.tif]
